# Health status of Polish children and adolescents after cancer treatment

**DOI:** 10.1007/s00431-017-3066-x

**Published:** 2017-12-22

**Authors:** Maryna Krawczuk-Rybak, Anna Panasiuk, Teresa Stachowicz-Stencel, Małgorzata Zubowska, Jolanta Skalska-Sadowska, Dorota Sęga-Pondel, Aneta Czajńska-Deptuła, Dorota Sławińska, Wanda Badowska, Elżbieta Kamieńska, Aneta Pobudejska-Pieniążek, Maria Wieczorek

**Affiliations:** 1 Department of Pediatric Oncology and Hematology, Medical University of Bialystok, Białystok, Poland; 2 Department of Pediatrics, Hematology and Oncology, Medical University of Gdansk, Gdansk, Poland; 3 Department of Pediatrics, Oncology, Hematology and Diabetology, Medical University of Lodz, Lodz, Poland; 4 Department of Pediatric Oncology, Hematology and Transplantology University of Medical Sciences, Poznan, Poland; 5 Department of Bone Marrow Transplantation, Oncology and Hematology, Medical University of Wroclaw, Wroclaw, Poland; 6 Department of Pediatric Oncology, Children’s Memorial Health Institute, Warsaw, Poland; 7 Department of Hematology, Oncology and Pediatric Bone Marrow Transplantation, Medical University of Lublin, Lublin, Poland; 8 Department of Pediatric Oncology and Hematology, County Hospital, Olsztyn, Poland; 9 Department of Pediatrics, Hematology and Oncology, Medical University of Szczecin, Szczecin, Poland; 10 Department of Pediatrics, Hematology and Oncology, Medical University of Zabrze, Zabrze, Poland; 11 Center of Pediatrics and Oncology by Dr E. Hanke, Chorzow, Poland

**Keywords:** Adolescents, Adults, Childhood cancer survivors, Epidemiology, Late effects

## Abstract

In the last 40 years, considerable progress was made in the treatment of childhood cancer. Nearly 80% of children achieve long-term clinical remission or are permanently cured. This improvement is however not without sacrifice. This is the first Polish study analyzing the general health status and epidemiology of organ late effects in the cohort of Polish childhood and adolescent cancer survivors monitored by doctors and registered in the on-line national database for late effects (*N* = 1761). This tool collects information on previous therapy and current health status (medical history, physical examination, laboratory tests) of cancer survivors. The survivors are invited to take part in the follow-up examination 5 years after the end of treatment. In the study group, 207 survivors (11.75%) had no complaints; whereas in 1554 cases (88.25%), one or more symptoms/complaints suggesting organ dysfunction were reported. In the whole group, the circulatory problems were most common (31.7%); more than 20% of survivors presented complaints or abnormal function of the urinary tract and had skin, dental, skeletal/muscular problems, or difficulty with chewing. Obesity or short stature alone (21.4%) and a variety of endocrine problems (short stature, obesity, thyroid dysfunction, and gonads toxicity) were present in 323 patients (118 females 15.0% and 205 males 21.0%). Gonadal dysfunction, as the only problem, occurred in 75 girls (9.6%) and 131 boys (13.4%). In our cohort, severe or life-threatening health conditions (3 and 4 grade according to toxicity criteria) were present in low percentage, i.e., 0.2% in the circulatory system, 0.3% in the respiratory tract and, 0.7% in kidney insufficiency.

*Conclusion*: Our findings indicate that many childhood cancer survivors demonstrate numerous complaints, even a short time after treatment, suggesting the importance of regular follow-up examinations in subsequent years.

**Table:** 

**What is Known:** • *Contemporary studies indicate that a significant number of childhood cancer survivors present different long-term side effects which influence their quality of life.*
**What is New:** • *This is the first nationwide study performed in the largest cohort of Polish childhood cancer survivors concerning general health status and frequency of organ dysfunction.*

## Introduction

In the last 40 years, we have observed progress in the treatment of different cancers during childhood—nearly 80% of children achieve long-term clinical remission or are permanently cured. This improvement is a result of more precise diagnosis (progress in radiology, molecular examinations), advances in chemotherapy, surgery, radiotherapy, and supportive care. In the growing population of childhood cancer survivors (CCS) (there are 300,000–500,000 CCS in Europe), we can find a wide range of late effects that appear after complex anticancer therapy—deterioration of life quality, late morbidity and mortality, dysfunction of different organs, endocrinopathies, and second cancers [9, 15, 24, 25].

Research into the health status and quality of life in the cohorts of CCS is conducted in many Western countries [9, 34]. In 2005, a group for late effects monitoring was formed within the Polish Society of Pediatric Oncology and Hematology; between 2006 and 2007, a platform was established as a website tool for CCS registration, which collects data on CCS from all over Poland; finally, in 2008, the Ministry of Health accepted this program, approving the structure, aims and organization of care, and health monitoring for CCS. The frequency of visits in the outpatient clinic depends on the type of cancer and aggressiveness of treatment.

## Purpose

We analyzed the general health status and epidemiology of organ late effects in the cohort of Polish CCS, taking into consideration diagnosis, type of therapy, age, sex, and time interval between the end of treatment and a follow-up, as well as demand of special care for survivors.

## Material and methods

After the end of the therapy, each patient should receive complex information for their general practitioner (or for specialists) concerning the history of illness, treatment, acute side effects, and guidelines for future follow-up. This information is prepared by the attending physician. The monitoring starts 2 years after the end of treatment simultaneously with cancer remission monitoring and is performed by the attending physician. The “actual” follow-up starts 5 years after the treatment. This task is delegated to the dedicated physician working in the outpatient setting, who has a direct contact with the attending physician and a complete insight into the patient’s medical history.

The frequency of follow-up visits depends on the type of cancer and its therapy—from minimum once a year for survivors with high probability of side effects to once every 2–5 years for patients who received less aggressive treatment. CCS are followed in outpatient clinics in children’s hospitals until they are 18 years old; sometimes, however rarely, up to the age of 24 years or even longer. At the beginning of the visit, every patient receives a detailed questionnaire on the present complaints, referring to every system/organ in the body. The application of additional investigations depends partly on the type of diagnosis and mostly on the previous treatment (e.g., echocardiogram for patients receiving anthracyclines, audiogram for survivors treated with platin-based agents or irradiation to the central nervous system, flow-cytometry for patients after hematopoietic stem cell transplantation, abdominal ultrasound for solid tumors located in the abdomen, and spirometry if there was surgery within the lung tissue or chest wall). No official international guidelines were used in our patients, but rather the recapitulation of the already published studies that were gathered into a logical plan for the respective survivor [21]. The visit ends with the verification of the complaints marked in the questionnaire and interpretation of the laboratory and additional test findings. On-line database is then filled in by a physician who follows survivors and registers the information on the previous therapy and current health status.

Analysis was performed in the cohort of 1761 registered CCS from 2016, who had complete datasets in the web-based tool. When the child visited the outpatient clinic for follow-up more than once, the results from the last visit were taken into consideration. This registry collects data on the medical history of disease and treatment, current medical history, physical examination and results of laboratory findings, radiological examinations, and functional investigations, allowing the analysis of the function of all organs and general health status of the survivors. The necessity to perform different analysis, e.g., echocardiography, audiometry, spirometry, and hormonal analysis, depends on risk-based recommendations.

The description of the health condition was classified according to the Common Terminology Criteria for Adverse Events (CTCAE) as the following: grade 1 (mild), grade 2 (moderate), grade 3 (severe), and grade 4 (serious/life-threatening) [33].

Typical statistical tests were used to calculate the number of different pathologies in the subgroups of the study patients. For comparison of the non-continuous data, chi-square test was used. STATA 2011 program was applied for statistical analysis.

The characteristics of the cohort of cancer survivors are presented in Table 1.

**Table 1:** Characteristics of Polish childhood cancer survivors cohort

	*N*	%
Sex		
Male	976	55.42
Female	785	44.58
Age at diagnosis		
0–1 years	145	7.75
2–4 years	725	38.75
5–9 years	450	24.05
10–14 years	354	18.92
15–18 years	197	10.53
Time from the end of treatment		
< 2 years	156	8.86
2–4 years	625	35.49
5–10 years	764	43.38
11–15 years	195	11.07
> 15 years	21	1.19
Age at the time of study		
< 5 years	50	2.84
5–10 years	347	19.70
11–15 years	553	31.40
15–20 years	610	34.64
> 20 years	201	11.41
Primary diagnosis		
Acute lymphoblastic leukemia	621	35.26
Acute myeloid leukemia	53	3.01
Hodgkin’s lymphoma	305	17.32
Non-Hodgkin’s lymphoma	126	7.16
Central nervous system tumors	64	3.63
Neuroblastoma	118	6.70
Wilms tumors	154	8.75
Soft tissue sarcomas	111	6.30
Bone tumors	74	4.20
Germ cell tumors	61	3.46
Hepatoblastoma	24	1.36
Retinoblastoma	31	1.76
Others (chronic myeloid leukemia, Langerhans histiocytosis, melanoma)	19	1.08
Types of the treatment		
Chemotherapy	594	33.73
Chemotherapy and radiotherapy	493	28.0
Chemotherapy and surgery	426	24.19
Chemotherapy, radiotherapy, and surgery	231	13.12
Surgery	17	0.97

HSCT (hematopoietic stem cell transplantation) was performed in 132 (7.50%) patients (in leukemias and solid tumors after megachemotherapy)

High percentage of patients after treatment for Hodgkin’s lymphoma (HL) (17.32%) was due to the fact that we started the follow-up from this group of patients in the initial period of database functioning. Epidemiological data show that in Poland patients treated for HL account for approximately 7%.

On the other hand, lower (3.63%) percentage of patients after brain tumors is due to their registration in separate database.

## Results

The following types of anticancer therapy were used in the study group: chemotherapy in 594 cases (33.73%), surgery alone in 17 cases (0.97%), chemotherapy and radiotherapy in 493 cases (24.19%), and chemo-, radiotherapy, and surgery in 231 cases (13.12%); additionally, bone marrow transplantation procedure was conducted in 132 cases (7.5%) (Table 1).

Normal function of all organs without complaints was observed in 207 (11.75%) survivors; whereas, in 239 (13.57%) cases, we found at least one symptom or complaint suggesting organ dysfunction, in 274 (15.56%)—dysfunction of two organs occurred, in further 249 (14.14%) three organs, and in 792 (44.97%)—four or more organs were affected.

In the whole group, the circulatory problems were observed most frequently (31.7%). More than 20% of the survivors presented complaints or abnormal function of the urinary tract (28.5%), skin problems (43.5%), dental problems or difficulty with chewing (26.6%), skeletal/muscular problems (23.5%), gastrointestinal problems (23.7%), obesity or short stature (21.4%), gonadal dysfunction (male) (25.7%), and immunologic disturbances (23.8%). Endocrine problems (short stature, obesity, thyroid dysfunction, and gonadotoxicity) were present in 323 patients (118 females, 15.0% and 205 males, 21.0%). Dysfunction of the gonads, as the only problem, occurred in 75 girls (9.6%) and 131 (13.4%) boys.

### Sex

**Fig. 1:**
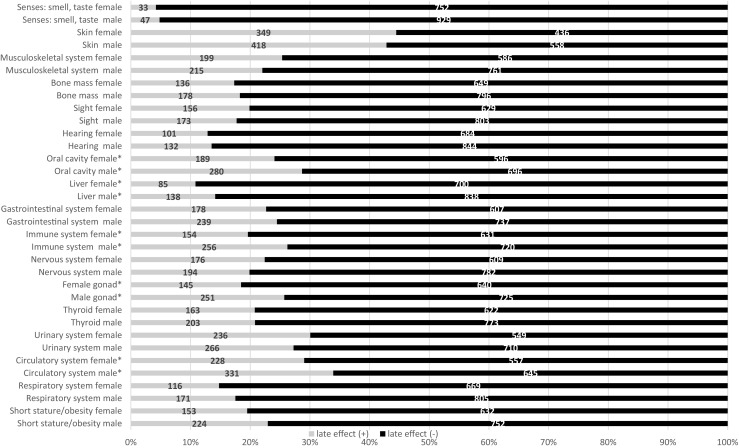
The frequency of different organ dysfunctions according to sex

The frequency of different organ dysfunctions according to sex is presented in Fig. 1. A higher frequency of complaints or organ abnormalities was observed in males than in females in the circulatory system (*p* = 0.029), the gonads (*p* < 0.0001), the immune system (*p* < 0.0001), the liver (*p* = 0.038), and dentition/the masticatory system (*p* < 0.0001).

### Age at diagnosis

A higher frequency of late effects was found in survivors diagnosed at younger age; the circulatory and immune system abnormalities were observed most often in children diagnosed between the age of 1 and 4 years (35%, *p* < 0.0001 and 27.3%, *p* = 0.005, respectively). The same tendency was noted for dental problems/difficulty with chewing (33%, *p* < 0.0001); whereas, infants were most frequently diagnosed with hearing problems (20.6%, *p* < 0.0001) and vision problems (22.9%, *p* = 0.012; Table 2). The thyroid gland dysfunction was present most frequently in children diagnosed between the age of 5 and 9 years (24. 5%, *p* = 0.001).

**Table 2:** Organ and systems toxicities according to age at diagnosis

System/organ	Age at the time of diagnosis					*p* value
	< 1 *N* = 145	1–4 *N* = 725	5–9 *N* = 450	10–14 *N* = 354	> 15 *N* = 197	
Short stature/obesity	24 (18.3%)	164 (23.5%)	92 (21.4%)	64 (19.9%)	33 (18.0%)	0.372
Respiratory system	18 (13.75)	115 (16.5%)	77 (17.9%)	45 (14.0%)	32 (17.5%)	0.572
Circulatory system	17 (13.0%)	244 (35.0%)	143 (33.3%)	102 (31.8%)	53 (29.0%)	< 0.0001
Urinary system	37 (28.2%)	215 (30.8%)	119 (27.7%)	92 (28.7%)	39 (21.3%)	0.155
Thyroid	9 (6.9%)	144 (20.7%)	105 (24.5%)	68 (21.2%)	40 (21.9%)	0.001
Male gonad	12 (17.4%)	98 (25.6%)	64 (22.8%)	41 (23.7%)	36 (36.0%)	0.077
Female gonad	8 (12.9%)	54 (17.2%)	40 (22.5%)	24 (16.2%)	19 (22.9%)	0.279
Nervous system	33 (74.8%)	154 (22.1%)	102 (23.8%)	59 (18.4%)	22 (12.0%)	0.007
Immune system	32 (24.4%)	190 (27.3%)	105 (24.5%)	64 (19.9%)	28 (15.3%)	0.005
Gastrointestinal system	25 (19.1%)	186 (26.7%)	100 (23.3%)	70 (21.8%)	36 (11.2%)	0.121
Liver	13 (9.9%)	85 (12.2%)	58 (13.5%)	44 (13.7%)	23 (12.6%)	0.803
Oral cavity/problems with chewing	40 (30.5%)	230 (33.0%)	105 (24.5%)	61 (19.0%)	33 (18.0%)	< 0.0001
Hearing	27 (20.6%)	119 (17.1%)	46 (10.7%)	27 (8.4%)	14 (7.7%)	< 0.0001
Sight	30 (22.9%)	150 (21.5%)	73 (17.0%)	55 (17.1%)	21 (11.5%)	0.012
Bone mass	21 (16.0%)	161 (23.1%)	108 (25.2%)	88 (27.4%)	36 (19.7%)	0.018
Musculo-skeletal system	11 (8.4%)	129 (18.5%)	89 (20.7%)	58 (18.1%)	27 (14.8%)	0.061
Skin	79 (39.7%)	389 (44.2%)	222 (48.3%)	192 (40.2%)	113 (38.3%)	0.08
Senses: smell, taste	9 (6.9%)	38 (5.5%)	18 (4.2%)	13 (4.0%)	2 (1.1%)	0.082

### Time of follow-up

The group of long-term survivors (more than 15 years) was not very numerous (*N* = 21). Those who ended their treatment between 5 and 10 years to the follow-up showed more late effects in the immune system (*p* < 0.0001), male gonads (*p* = 0.003), and dentition/the masticatory system (*p* = 0.002). The highest incidence of complaints/symptoms was found 11 to 15 years after the treatment completion: in the circulatory system (*p* < 0.0001), the respiratory system (*p* = 0.024), the thyroid gland (*p* < 0.006), the digestive tract (*p* < 0.0001), and the nervous system (*p* < 0.0001) (Table 3).

**Table 3:** Organ and systems toxicities according to the follow-up time

System/organ	Time of follow-up					*p* value
	< 2 *N* = 156	2–4 *N* = 625	5–10 *N* = 764	11–15 *N* = 195	> 15 *N* = 21	
Short stature/obesity	30 (19.2%)	115 (18.4%)	180 (23.6%)	45 (23.1%)	7 (33.3%)	0.092
Respiratory system	17 (10.9%)	87 (13.9%)	139 (18.2%)	41 (21.0%)	3 (14.3%)	0.024
Circulatory system	25 (16.0%)	162 (25.9%)	290 (38.0%)	75 (38.5%)	7 (33.3%)	< 0.0001
Urinary system	30 (19.2%)	144 (23.0%)	250 (32.7%)	68 (34.9%)	10 (47.6%)	< 0.0001
Thyroid	19 (12.2%)	129 (20.6%)	208 (27.2%)	56 (28.7%)	5 (23.8%)	< 0.0001
Male gonad	21 (25.3%)	63 (18.6%)	128 (29.2%)	0	0	0.003
Female gonad	11 (15.1%)	37 (12.9%)	69 (21.2%)	26 (28.9%)	2 (20.0%)	0.006
Nervous system	16 (10.3%)	105 (16.8%)	194 (25.4%)	50 (25.6%)	5 (23.8%)	< 0.0001
Immune system	25 (16.0%)	133 (21.3%)	213 (27.9%)	43 (22.1%)	5 (23.8%)	< 0.0001
Gastrointestinal system	19 (12.2%)	129 (20.6%)	208 (27.2%)	56 (28.7%)	5 (23.8%)	< 0.0001
Liver	11 (7.1%)	49 (7.8%)	111 (14.5%)	41 (21.0%)	11 (52.4%)	< 0.0001
Oral cavity/problems with chewing	31 (19.9%)	143 (22.9%)	235 (30.8%)	57 (29.2%)	3 (14.3%)	0.002

### Diagnosis

In the most numerous group of survivors, after treatment for acute lymphoblastic leukemia (ALL), more than 25% of patients presented symptoms/complaints suggesting the following problems: cardiac (38.8%), digestive (26.4%), immune (31.1%), dental/difficulty with chewing (28.5%), male gonadal dysfunction (26.3%), and skin problems (44.8%). Patients treated for AML most often demonstrated cardiac (35.8%), immune (34.0%), respiratory (26.4%), female gonadal (38.5%), growth (34.0%), neurological (26.4%), and dental (30.2%) problems (Table 4).

**Table 4:** Organ and systems toxicities according different diagnoses

System/organ	Diagnosis			
	ALL *N* = 621	AML *N* = 53	HL *N* = 305	NHL *N* = 126
Short stature/obesity	147 (23.7%)	18 (34.0%)	47 (15.4%)	26 (20.6%)
Respiratory system	119 (19.2%)	14 (26.4%)	47 (15.4%)	14 (11.1%)
Circulatory system	241 (38.8%)	19 (35.8%)	82 (26.9%)	42 (33.3%)
Urinary system	153 (24.6%)	15 (28.3%)	55 (18.0%)	29 (23.0%)
Thyroid	112 (18.0%)	11 (20.8%)	84 (27.5%)	25 (19.8%)
Male gonad	92 (26.3%)	5 (18.5%)	46 (25.1%)	19 (21.8%)
Female gonad	39 (14.4%)	10 (38.5%)	17 (13.9%)	4 (10.3%)
Nervous system	130 (20.9%)	14 (26.4%)	28 (9.2%)	17 (13.5%)
Immune system	193 (31.1%)	18 (34.0%)	66 (21.6%)	27 (21.4%)
Gastrointestinal system	164 (26.4%)	8 (15.1%)	63 (20.7%)	29 (23.0%)
Liver	89 (14.3%)	7 (13.2%)	37 (12.1%)	19 (15.1%)
Oral cavity/problems with chewing	177 (28.5%)	16 (30.2%)	59 (19.3%)	25 (19.8%)
Hearing	56 (9.0%)	5 (9.4%)	17 (5.6%)	7 (5.6%)
Sight	122 (19.6%)	13 (24.5%)	33 (10.8%)	10 (7.9%)
Bone mass	155 (25.0%)	7 (13.2%)	30 (9.8%)	17 (13.5%)
Musculo-skeletal system	146 (23.5%)	9 (17.0%)	37 (12.1%)	13 (10.3%)
Skin	278 (44.8%)	27 (50.9%)	100 (32.8%)	46 (63.5%)
Senses: smell, taste	35 (5.6%)	2 (3.8%)	5 (1.6%)	0
System/organ	Diagnosis			
	CNS tumors *N* = 64	NBL *N* = 64	Wilms tumors *N* = 154	STS *N* = 111
Short stature/obesity	28 (43.8%)	31 (26.3%)	20 (13.0%)	21 (18.9%)
Respiratory system	10 (15.6%)	23 (19.5%)	17 (11.0%)	15 (13.5%)
Circulatory system	25 (39.1%)	24 (20.3%)	41 (26.6%)	32 (28.8%)
Urinary system	29 (45.3%)	39 (33.1%)	83 (53.9%)	38 (34.2%)
Thyroid	32 (50%)	21 (17.8%)	22 (14.3%)	27 (24.3%)
Male gonad	29 (45.3%)	14 (24.1%)	9 (12.0%)	22 (32.4%)
Female gonad	35 (40.0%)	15 (25.0%)	12 (15.2%)	11 (25.6%)
Nervous system	47 (73.4%)	34 (28.8%)	33 (21.4%)	23 (20.7%)
Immune system	9 (14.1%)	32 (27.1%)	34 (22.1%)	18 (16.2%)
Gastrointestinal system	20 (31.3%)	32 (27.1%)	38 (24.7%)	18 (16.2%)
Liver	10 (15.6%)	12 (10.2%)	19 (12.3%)	12 (10.8%)
Oral cavity/problems with chewing	28 (43.8%)	39 (33.1%)	46 (29.9%)	38 (34.2%)
Hearing	34 (53.1%)	32 (27.1%)	22 (14.3%)	22 (19.8%)
Sight	35 (54.7%)	18 (15.3%)	74 (48.1%)	27 (24.3%)
Bone mass	15 (23.4%)	16 (13.6%)	18 (11.7%)	16 (14.4%)
Musculo-skeletal system	29 (45.3%)	28 (23.7%)	40 (26.0%)	33 (29.7%)
Skin	31 (48.4%)	53 (44.9%)	74 (48.1%)	55 (49.5%)
Senses: smell, taste	19 (29.7%)	7 (5.9%)	4 (2.6%)	4 (3.6%)
System/organ	Diagnosis			
	Bone tumors *N* = 74	Germ cell tumors *N* = 61	HBL *N* = 24	RBL *N* = 31
Short stature/obesity	14 (18.9%)	9 (14.8%)	4 (16.7%)	9 (29.0%)
Respiratory system	17 (23.0%)	7 (11.5%)	2 (8.3%)	1 (3.2%)
Circulatory system	28 (37.8%)	15 (24.6%)	6 (25.0%)	2 (6.5%)
Urinary system	22 (29.7%)	22 (36.1%)	8 (33.3%)	2 (6.5%)
Thyroid	10 (13.5%)	12 (19.7%)	3 (12.5%)	3 (9.7%)
Male gonad	8 (28.6%)	19 (61.3%)	3 (16.7%)	0
Female gonad	8 (17.4%)	9 (30.0%)	0	2 (11.8%)
Nervous system	20 (27.0%)	9 (14.8%)	4 (16.7%)	5 (16.1%)
Immune system	6 (8.1%)	10 (16.4%)	2 (8.3%)	2 (6.5%)
Gastrointestinal system	15 (20.3%)	17 (27.9%)	9 (37.5%)	3 (9.7%)
Liver	4 (5.4%)	8 (13.1%)	2 (8.3%)	2 (6.5%)
Oral cavity/problems with chewing	11 (14.9%)	12 (19.7%)	7 (41.2%)	5 (16.1%)
Hearing	9 (12.2%)	17 (27.9%)	8 (33.3%)	1 (3.2%)
Sight	6 (8.1%)	7 (11.5%)	2 (8.3%)	28 (90.3%)
Bone mass	23 (31.1%)	9 (16.4%)	0	3 (9.7%)
Musculo-skeletal system	62 (83.8%)	10 (16.4%)	1 (4.2%)	2 (6.5%)
Skin	55 (74.3%)	33 (54.1%)	9 (37.5%)	1 (3.2%)
Senses: smell, taste	0	3 (4.9%)	0	0

*ALL* acute lymphoblastic leukemia, *AML* acute myeloid leukemia, *HD* Hodgkin’s lymphoma, *NHL* non-Hodgkin’s lymphoma, *CNS* central nervous system, *NBL* neuroblastoma, *STS* soft tissue sarcomas, *HBL* hepatoblastoma, *RBL* retinoblastoma

In the group of HL survivors, impaired cardiac function (26/9%), thyroid (27/5%), male gonadal (25/1%), and skin problems (32/8%) were most common, whereas after non-Hodgkin’s lymphoma—complaints from the circulatory system (33.3%) (Table 4).

Survivors of the CNS tumors demonstrated very high frequency of disturbances affecting almost all organs, with the highest frequency of short stature/obesity (43.8%), nervous system (73.4%), vision (54.7%), hearing (53.1%), dentition/masticatory system (43.8%), circulatory system (39.1%), urinary system (45.3%), thyroid gland (50.0%), gonads (male 45.3%, female 40%), skeletomuscular system (45.3%), and skin (48.4%).

The treatment for neuroblastoma deteriorated most often the function of the urinary system (33.1%), the nervous system (28.8%), the digestive and immune systems (27.1%), dentition/masticatory system (33.1%), and short stature (26.3%).

After the treatment for Wilms tumor, most frequent were the symptoms/complaints of the urinary tract (53.9%), cardiac (26.6%), dental/chewing (29.9%), affecting vision (48.1%), and the skeletal/muscular ones (26%). After soft tissue sarcomas, symptoms from the circulatory system (28.8%), the urinary tract (34.2%), and gonadal dysfunction (32.4% male, 25.6% female) were most common. Bone cancer survivors most frequently presented skeletal/muscular dysfunction (83.8%), circulatory problems (37.8%), skin problems (74.3%), low bone mass (31.1%), urinary tract problems (29.7%), male gonadal problems (28.6%), and symptoms from the nervous system (27%). Gonadal (male 61.3% and female 30%) and urinary dysfunction (36.1%), hearing problems (27.9%), cardiac (24.6%), and gastrointestinal (27.9%) complaints were found in patients treated for germ cell tumors. Interestingly, the survivors of hepatoblastoma rarely presented liver dysfunction (8.3%); whereas, 25%—cardiac problems, 37.5%—symptoms from the digestive tract, 41.2%—dental/chewing problems, and 33%—hearing dysfunction. Retinoblastoma survivors in 90.3% had visual problems and 29% had short stature or were obese (Table 4).

When we took into consideration the type of anticancer therapy, the survivors treated with chemotherapy alone had a lower number of different complaints; whereas, those who underwent hematopoietic stem cell transplantation presented the highest number of problems from different organs, among which the most frequent were male and female infertility (47.4 and 44.6%, respectively), thyroid insufficiency (33.3%), skin discoloration or chronic graft versus host disease (44.7%), and low immunoglobulin levels or disproportion in the subclasses of lymphocytes (32.6%) (Table 5). In every treatment group, cardiac symptoms were found in over 30% of the survivors, followed by skin, immune, and gastrointestinal problems (Table 5).

**Table 5:** Organ and systems in different types of treatment

System/organ	Types of treatment			
	Chemotherapy only *N* = 594	Chemotherapy and radiotherapy *N* = 493	Chemotherapy, radiotherapy, and surgery *N* = 231	HSCT *N* = 132
Short stature/obesity	131 (22.1%)	104 (21.1%)	67 (29.0%)	50 (37.9%)
Respiratory system	107 (18.0%)	85 (17.2%)	36 (15.6%)	26 (19.7%)
Circulatory system	205 (34.5%)	173 (35.1%)	71 (30.7%)	44 (33.3%)
Urinary system	135 (22.7%)	113 (22.0%)	104 (45.0%)	45 (34.1%)
Thyroid	101 (17.0%)	131 (26.6%)	70 (30.3%)	44 (33.3%)
Male gonad	69 (21.4%)	90 (30.2%)	36 (29.3%)	36 (47.4%)
Female gonad	40 (14.8%)	33 (16.9%)	31 (28.7%)	25 (44.6%)
Nervous system	104 (17.5%)	89 (18.1%)	82 (35.5%)	39 (29.5%)
Immune system	174 (29.3%)	134 (27.2%)	41 (17.7%)	43 (32.6%)
Gastrointestinal system	143 (24.1%)	115 (23.3%)	59 (25.5%)	36 (28.8%)
Liver	80 (13.5%)	69 (14.0%)	29 (12.6%)	19 (14.4%)
Oral cavity/problems with chewing	162 (27.3%)	118 (23.9%)	81 (35.1%)	33 (25.0%)
Hearing	47 (7.9%)	43 (8.7%)	68 (29.4%)	23 (17.4%)
Sight	101 (17.0%)	88 (17.8%)	68 (29.4%)	27 (20.5%)
Bone mass	127 (21.4%)	85 (17.2%)	49 (21.2%)	36 (27.3%)
Musculo-skeletal system	119 (20.0%)	86 (17.4%)	79 (34.2%)	39 (29.5%)
Skin	251 (42.3%)	186 (37.7%)	125 (54.1%)	59 (44.7%)
Senses: smell, taste	28 (4.7%)	14 (2.8%)	24 (10.4%)	5 (3.8%)

*HSCT* hematopoietic stem cell transplantation

Taking into consideration CTCAE for the description of health condition, 99.7% of the survivors presented mild (1) and moderate (2) grade sequelae and 0.30% severe (3) or serious (4) grade of respiratory tract dysfunction. Grades 1 and 2 symptoms/complaints from the circulatory system were found in 99.9% and grades 3 and 4 in 0.1%. Symptoms from the urinary tract in 1 and 2 grades were observed in 96.42% and in grade 3 or 4 in 3.58%. Thyroid dysfunction in grade 1 or 2 was present in 99.29%, in grade 3 or 4 in 0.71%.

After the end of treatment, the largest group of survivors was followed-up in pediatric oncology outpatient clinic—over 70%. Nearly 20% were followed-up in endocrinological outpatient clinic due to thyroid or gonadal disorders. The smallest number participated in follow-up in gastrological or/and liver diseases outpatient clinics (5.3%).

## Discussion

The first reports from the British CCSS were based on a cohort of 17,981 individuals diagnosed with childhood cancer between 1940 and 1991, who survived at least 5 years. Other large registries were created in some European countries, the USA and Canada. In 2011, there were nearly 388,501 CCS in the USA, of whom 83.5% were ≥ 5 years post-diagnosis [4, 25, 27, 28, 34].

The results of anticancer treatment of Polish children have also changed in the last 40 years. Currently, nearly 80% of children with cancer achieve long-time clinical remission or are cured. In the USA or Great Britain, the observation of survivors started as Childhood Cancer Survivors Studies (CCSS) many years ago; they are focused on physical and psychological late effects, such as organ dysfunction, second neoplasms, endocrine problems, or mental and social consequences [23, 27, 34]. Unfortunately, there are no such investigations in Central and Eastern Europe.

This is the first nationwide study performed in the largest cohort of Polish CCS. The arrangement of the Polish follow-up guidelines is similar to the Scottish Intercollegiate Guidelines Network (SIGN): the frequency of visits in outpatient clinics and range of examinations depend on diagnosis, previous anticancer treatment, and the risk of late effects [30]. Generally, the follow-up of survivors over 18 years old has to be carried out in the outpatient clinics for adults. Therefore, our study group consists mainly of children and adolescents, and only a small number of young adults. Moreover, in other countries, the long-term observations were started with the cohorts diagnosed in 1970s or even earlier [28, 34]; whereas, our study period is short and the population is young. The strength of our study is that the observations were based on physicians’ interviews and reports (history and physical examination), biochemical, hormonal testing, and radiological examinations. We used not only patient self-completion questionnaires. The information given by patients was verified at the end of each follow-up visit, the reason for marking a respective complaint was made clear and only confirmed complaints appeared in the database. If the examinations performed ruled out the reason for the complaint, test results were also added to the database. Finally, information concerning the referral to a specialist, if the complaints were true, was marked. We found that only 11.75% of the survivors were in very good general health, without complaints or signs of any organ dysfunction, whereas 29.13% demonstrated. Complaints or symptoms of dysfunction of one or two organs and nearly 45% presented the signs of abnormal function of four or more organs. The American multi-institutional CCSS based on interviews and questionnaires, including 10.397 adult CCS, reported that 62.3% had at least one, 37.6% at least two, and 23.8% multiple chronic health conditions [25]. In the Dutch cohort (1.315 survivors), 70% presented at least one chronic health condition, including 40% with severe or disabling condition [11]. A Japan nationwide study reported that 56% of young CCS (mean age 23.6 years) experienced at least one late effect. A similar observation was reported from Korea (59.8%) [13, 26]. In our cohort, severe or life-threatening health conditions (3 and 4 grade according to CTCAE) were rare—from 0.2% for the circulatory system, 0.3% for the respiratory tract, and 0.7% for kidney insufficiency. Analysis made in Korea in a younger population showed severe late effects in 10.8% of survivors [13]. In another Dutch study, higher frequency of severe late effects was observed in older survivors, after very long follow-up time (> 20 years, 47.8% patients), as compared to a shorter follow-up time (< 20 years, 27.9%) [6]. In the American cohort, the incidence of severe life-threatening conditions or death caused by a chronic condition occurred in 27.5% survivors at the mean age of 26.6 years; whereas, late sequelae were present in 42.4% of survivors at the median of 30 years after cancer diagnosis [25]. The results from Southern California showed that 15 years after diagnosis, nearly 40% of survivors developed at least one chronic condition [7]. The age at follow-up and the time after therapy completion seem to be very important; organ senescence, faster tissue aging after chemo- and radiotherapy, health behaviors, and differences in anticancer treatment exert their effects. Observations from different studies have indicated a growing prevalence and severity with time after diagnosis [7], which has not achieved plateau [25]. Amstrong et al. showed that a group of 24-year-old survivors of childhood cancer had the same cumulative incidence of grades 3 to 5 health conditions as their 50-year-old siblings. It was especially visible in a group of survivors older than 35 years, where nearly 20% experienced higher incidence of cardiovascular events and second cancers. The authors predict that 50% of childhood cancer survivors older than 50 years will have experienced severe diseases as a result of multiple organs dysfunction [3]. In the USA, in one of the latest studies, the prevalence of any chronic condition in survivors of more than 5 years ranged from 66% (in survivors aged 5 to 19 years) to 88% (in survivors aged 40–49 years) [27]. Our study revealed the highest incidence of cardiovascular problems (reported by 31.7%) and endocrinological problems (18.3%). They were especially visible in patients after most aggressive treatment, such as bone marrow transplantation or after complex treatment including radiotherapy. In comparison with other reports, our study group was younger and the follow-up period shorter. However, even though the percentage of survivors with health problems is high, the degree of abnormalities is low (grade 1 or 2). In the studies conducted in other countries, based on older populations of survivors, cardiovascular problems are identified as one of the most serious and frequent complications leading to higher mortality [1, 2, 8, 9, 12, 16, 31, 32].

We found higher frequency of late effects in survivors diagnosed in younger age. The most common symptoms included circulatory, thyroid, neurological and immune abnormalities, dental problems/difficulty with chewing, hearing and vision problems, and osteopenia. In a study conducted by Han et al., where the median time after diagnosis was shorter (7.8 years), and the age at diagnosis and current age showed a positive correlation with the number of late effects [13]. The latest data from EUROCARE-5 focused on adolescents and young adults diagnosed between 2000 and 2007 exhibited improved survival in children and adolescents, but relatively poorer in adolescents and young adults than in children [31]. Similarly, in a Slovenian study, older age at diagnosis decreased the risk for late sequelae [10].

We observed higher frequency of complaints and symptoms of some organ dysfunctions (circulatory system, gonadotoxicity, hepatotoxicity, immune, and dental problems) in males. It remains in opposition to the results obtained by Oeffinger et al. in adult CCS, who found that females were more predisposed to have any adverse conditions, including 1.5 times more severe (3 or 4 grade) late effects [25]. American registries showed similar prevalence of chronic disorders in both sexes, from grade 1 or 2 to severe conditions, with a frequency increasing with age, especially in women [27].

A Korean study showed no differences between sexes in the frequency of late effects in younger CCS (mean age 14.2 years) [13]. Similarly, the results from Slovenia in younger CCS did not indicate the sex impact on the frequency of late effects [1], but suggest the effect of age at the follow-up and time after the end of treatment on the prevalence of chronic conditions. In our group, where the time of follow-up was not very long, the highest incidence of complaints/symptoms was observed in survivors who completed their treatment between 11 and 15 years of age (from the circulatory and respiratory systems, thyroid gland, female gonads, digestive tract, liver, and nervous system). According to Hudson et al., poor general status and chronic health conditions increase with age, particularly among females [18].

The type of anticancer therapy, especially most aggressive treatment (radiotherapy in complex treatment or megachemotherapy), seems to be most important. We observed a vast array of complaints and organ dysfunctions in these groups of survivors, especially after bone marrow transplantation. Other authors reported a large spectrum of serious chronic conditions and impairments involving different organs even in younger age [14]. In a Dutch study, severe adverse events were observed in 55% of survivors after radiotherapy and only in 15% after chemotherapy alone [11]. Poorer health status, physical condition, and quality of life have also been demonstrated in other studies [5, 13, 29]. Radiotherapy affects the function of all the exposed organs leading to endocrinopathies, mental and growth problems, obesity, pulmonary and cardiac complications, and second neoplasms [5, 18, 25]. This problem is especially known in older CCS, particularly in those treated with older techniques of irradiation [2, 11, 18, 19, 31], and contributes to changes in anticancer treatment during childhood, reducing indications for radiotherapy in early childhood. Phillips et al. defined the susceptibility to different chronic conditions and the magnitude of health challenges for CCS. Their analysis of results from the US population indicated high morbidity (70% mild or moderate chronic condition and 32% severe chronic condition) [27]. This results in earlier senesce and shorter life expectancy of CCS [17, 20].

In conclusion, our observations made in Poland in a group of survivors shortly after treatment completion showed that many survivors demonstrated numerous organ dysfunctions, thus indicating the necessity of regular follow-up examinations in subsequent years.

Taking into consideration that some of the presented late effects, even shortly after the end of treatment, can manifest and/or progress in the future, long-term monitoring is necessary for early detection of the symptoms of organ dysfunction. Our analysis indicates that most survivors and their parents receive basic information on health status, prophylaxis, and treatment at the time of treatment, as well at the time of transition to adulthood [22]. Similarly, adult primary healthcare providers and other specialists are informed about general late effects of anticancer treatment, although there is no official funding for special outpatient clinics dedicated to the CCS. In Poland, pediatric care covers patients up to the age of 18, and for childhood cancer survivors it can be extended up to the age of 24 years; after that age, the survivors make the transition to general practitioners and specialists (if need be). The most difficult problem is the organization of outpatient clinics and/or departments for adult childhood cancer survivors where they could receive adequate education and multidisciplinary care. Patient and doctor awareness and knowledge are essential for prophylaxis, early detection of adverse late effects, normal functioning, and good life quality. The development of effective model of care for young cancer survivors, transition program suitable for an individual survivor, continuation of the follow-up beyond early twenties, and care started in childhood seem to be most crucial for Polish childhood cancer survivors.

## Abbreviations

ALL: Acute lymphoblastic leukemia
AML: Acute myeloid leukemia
CCS: Childhood cancer survivors
CCSS: Childhood Cancer Survivors Study
CTCAE: Common Terminology Criteria for Adverse Events
EUROCARE: European Cancer Registry
HL: Hodgkin’s lymphoma
SIGN: Scottish Intercollegiate Guidelines Network
